# In vivo role of different domains and of phosphorylation in the transcription factor Nkx2-1

**DOI:** 10.1186/1471-213X-11-9

**Published:** 2011-02-23

**Authors:** Daniel Silberschmidt, Alina Rodriguez-Mallon, Prathiba Mithboakar, Gaetano Calì, Elena Amendola, Remo Sanges, Mariastella Zannini, Marzia Scarfò, Pasquale De Luca, Lucio Nitsch, Roberto Di Lauro, Mario De Felice

**Affiliations:** 1 Stazione Zoologica Anton Dohrn, Villa Comunale, 80121, Napoli, Italy; 2 IRGS, Biogem, Via Camporeale, 83031 Ariano Irpino (AV), Italy; 3 Dipartimento di Biologia e Patologia Cellulare e Molecolare, Università Federico II, Via Pansini 5, 80131 Napoli, Italy; 4 Institute of Experimental Endocrinology and Oncology "G. Salvatore", National Research Council, Via Pansini 5, 80131 Napoli, Italy

## Abstract

**Background:**

The transcription factor Nkx2-1 (also known as TTF-1, Titf1 or T/EBP) contains two apparently redundant activation domains and is post-translationally modified by phosphorylation. We have generated mouse mutant strains to assess the roles of the two activation domains and of phosphorylation in mouse development and differentiation.

**Results:**

Mouse strains expressing variants of the transcription factor Nkx2-1 deleted of either activation domain have been constructed. Phenotypic analysis shows for each mutant a distinct set of defects demonstrating that distinct portions of the protein endow diverse developmental functions of Nkx2-1. Furthermore, a mouse strain expressing a Nkx2-1 protein mutated in the phosphorylation sites shows a thyroid gland with deranged follicular organization and gene expression profile demonstrating the functional role of phosphorylation in Nkx2-1.

**Conclusions:**

The pleiotropic functions of Nkx2-1 are not all due to the protein as a whole since some of them can be assigned to separate domains of the protein or to specific post-translational modifications. These results have implication for the evolutionary role of mutations in transcription factors.

## Background

Transcription factors (TFs) bind to DNA and regulate mRNA synthesis in response to different stimuli via multiple protein domains endowing separate functions such as the binding to small ligands, the recognition of specific DNA sequences and the ability to activate or repress transcription. For the latter function it is frequently observed that more than one activation or repression domain can be present in a single TF [1]. In addition, it is well known that transcription factors can be regulated by post-translational modifications, chiefly phosphorylation[2]. However, the function of the diverse activation domains included in a single transcription factor or the role of post-translational modification has been assessed largely in cultured cells. This approach has obvious limitations since many TFs play important roles in diverse cell types and at different stages of development. Thus, whether the diverse functions of a TF could be assigned to separate protein domains or to post-translational modifications is a question that has been rarely addressed and it requires to carry out structure-function relationships studies in whole organisms expressing modified transcription factors lacking only one of its domains and/or mutated in its phosphorylation sites in order to block such post-translational modification. The homeodomain containing transcription factor Nkx2-1 (also called TTF-1, Nkx2-1 or T/EBP)[3] is well suited for this type of studies since it plays important roles in organogenesis and differentiation of several organs such as lung, brain, thyroid and pituitary[4]. In addition, it has been suggested that Nkx2-1 plays diverse roles at different stages in thyroid organogenesis, implying that it may change function, and, hence, target genes, during this process[5]. In keeping with this notion, thyroid specific ablation of the gene encoding Nkx2-1, carried out late in organogenesis, results in altered follicular organization of the thyroid gland[6], while knock-out of the same gene results in complete absence of the gland[4].

Nkx2-1 contains two well defined transcription activation domain that appear to be redundant for function in co-transfection assays[7]. Furthermore, Nkx2-1 is post-translationally modified by phosphorylation but both in DNA binding or in co-transfection assays no role could be assigned to this modification[8]. However, that phosphorylation might be important for Nkx2-1 transcriptional activity has been suggested in studies indicating that ERK-mediated phosphorylation of this transcription factor might play a role in Ras-induced loss of its transcriptional activity in an in vitro model of thyroid tumoral transformation[9]. Furthermore, studies in transgenic mice have demonstrated that mice homozygous for a *Nkx2-1* allele encoding a phosphorylation-resistent protein are defective for lung cell differentiation, but no data are available on the role of Nkx2-1 phosphorylation in other organs[10]. In this study we tested whether the two activation domains of Nkx2-1 have separate functions *in vivo *and demonstrate that these domains plays differential roles in thyroid and pitutary. Furthermore, we demonstrate that a *Nkx2-1 *mutant, encoding a phosphorylation defective protein, brings about critical defects in organization of thyroid follicles, without affecting earlier stages of development that are known to be dependent on the presence of the Nkx2-1 protein.

Taken together these data show that at least some of the multiple functions of Nkx2-1 are endowed into separate domains of the protein and that other functions depend on specific protein modifications. We believe that these data supporting evidence for mechanisms capable of generating novel functions in evolution.

## Results

### Generation of knock-in mice expressing Nkx2-1 mutants

**Figure 1:**
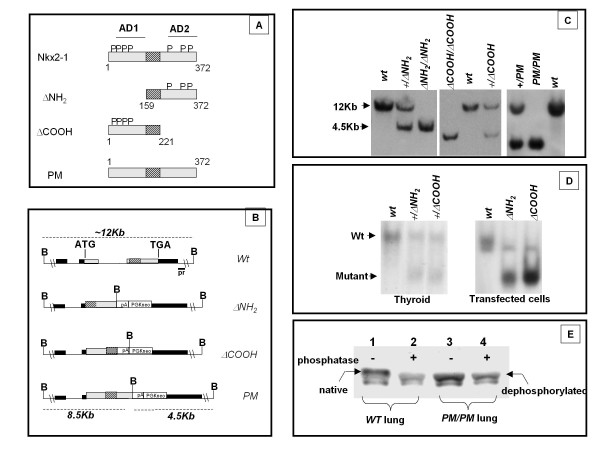
**Generation of mice carrying Nkx2-1 mutant alleles**. (**A**) The structure of the Nkx2-1 mutants is schematically shown. Numbering of amino acids is shown according to [19]. *P *indicates phosphorylated serine residues according to [8]; AD1 and AD2, activation domains. (**B**) Genomic structure of the *Nkx2-1 *locus, wild type allele and alleles modified by homologous recombination. *Black boxes *represent exons; *hatched box *the homeobox; ATG and TGA codons are indicated. The probe used for genotyping ES cell clones and mice is indicated by a black bar labeled *pr. PGKneo*, selection marker; *pA*, SV40 poly(A) sequence; *B*, *Bam*HI. (**C**) Southern blot analysis of genomic DNA from mouse tails digested with *Bam*HI and probed  probe within indicated in panel **B**. The lower band corresponds to the mutated allele (4.5 kb), the upper band to the wild type allele (12 kb). (**D**) Cellular extract from wild type and mutated mouse thyroids (left) were used in EMSA assays with an oligonucleotide containing a high affinity Nkx2-1 binding site. Extracts from FRTL-5 cells transfected with plasmids encoding mutated forms of Nkx2-1 were used as controls (right). Genotype of the mice and plasmids used in trasfected cells are indicated on each lane. (**E**) Lung homogenates (35 μg of protein) from wild type and PM/PM mice (E18.5) were phosphatase treated (+) or untreated (-), subjected to SDS PAGE, electrophoretically transferred to nitrocellulose and probed with anti Nkx2-1 antibody. The phosphate treatment increases the apparent mobility of wild type Nkx2-1 but does not affect the mobility of PM protein.

We previously demonstrated, by co-transfection assays carried out in non-thyroid cells in culture, that three *Nkx2-1 *mutants (Figure 1, panel A) encoding proteins in which either transactivation domain 1 or 2 was deleted (herein denominated ΔNH_2 _and ΔCOOH respectively) or phosphorylable serine residues have been mutagenized (herein denominated PM), all retained their transcriptional activities in vitro[7,8]. In order to assess the function of these mutants in the mouse, we generate three novel mouse strains, each homozygous for alleles encoding either ΔNH_2_, ΔCOOH, or PM Nkx2-1 protein. A knock-in approach was designed to rearrange the *Nkx2-1 *locus (Figure 1, panel B) in such a way to exclude the synthesis of the wild type gene product and to direct the expression of the mutant allele under the regulatory regions of the endogenous gene. Appropriate homologous recombination was achieved with all constructs and the heterozygous mice produced were backcrossed for seven generation with C57BL/6J wt mice to obtain all mutant mice in the same genetic background. Heterozygous mice were then intercrossed to generate homozygous mice (Figure 1, panel C).

The expression of ΔNH_2 _or ΔCOOH mutant proteins in thyroid tissue was evaluated in a mobility shift assay carried out using an oligonucleotide containing a high affinity Nkx2-1 binding site (oligonucleotide C, see "Materials and Methods"). Protein extracts from either *+/ΔNH_2 _*or *+/ΔCOOH *heterozygous thyroids show two DNA binding activities consistent with the presence of both wild type (wt) and mutant proteins (Figure 1, panel D). The levels of expression of the Nkx2-1 mutants are comparable to those of wild type.

To verify that the rearranged *PM *allele was not producing any phosphorylated protein, western blot analysis was carried out on lung tissues dissected from E18 *PM/PM *embryos. Lambda phosphatase treatment of protein extract from wt lung results in an increase in the relative mobility of Nkx2-1 on SDS-PAGE (Figure 1, panel E, lane 1 vs. 2). The PM protein has a mobility identical of the phosphatase treated Nkx2-1 (Figure 1, panel E, lane 3 vs. 2) and shows no further increase in mobility after phosphatase treatment (Figure 1, panel E, lane 3 vs. 4). These data strongly suggest that PM Nkx2-1 is not phosphorylated in vivo, as already demonstrated in cell lines [8].

### Both activation domains and phosphorylation in the Nkx2-1 protein are essential for life

Mice heterozygous for mutant alleles encoding either ΔNH_2, _ΔCOOH or PM are born and develop normally, without apparent abnormalities in growth or reproduction. On the contrary, no live pups were obtained from mice homozygous for any of the three mutant alleles. These mice die at birth, presumably of respiratory failure. Necropsy of both *ΔNH_2 _*and *ΔCOOH *homozygous reveals severe lung abnormalities comparable with those described in *Nkx2-1 null *mice (data not shown); lungs do develop in *PM/PM *mice but they show severe functional anomalies, as already reported [10]. These results indicate that both transactivation domains of Nkx2-1 as well as its phosphorylation are required for normal development and differentiation of lungs.

Previous studies also demonstrated that at least one copy of a functional *Nkx2-1 *allele is required for the organogenesis of both pituitary and thyroid[4]. In order to address whether different domains of Nkx2-1 or its phosphorylation have specific roles in the development of these two structures, we decided to analyze in better detail the phenotype of both developing pituitary and thyroid in mutant embryos.

### Pituitary organogenesis in wild type and mutant embryos

**Figure 2:**
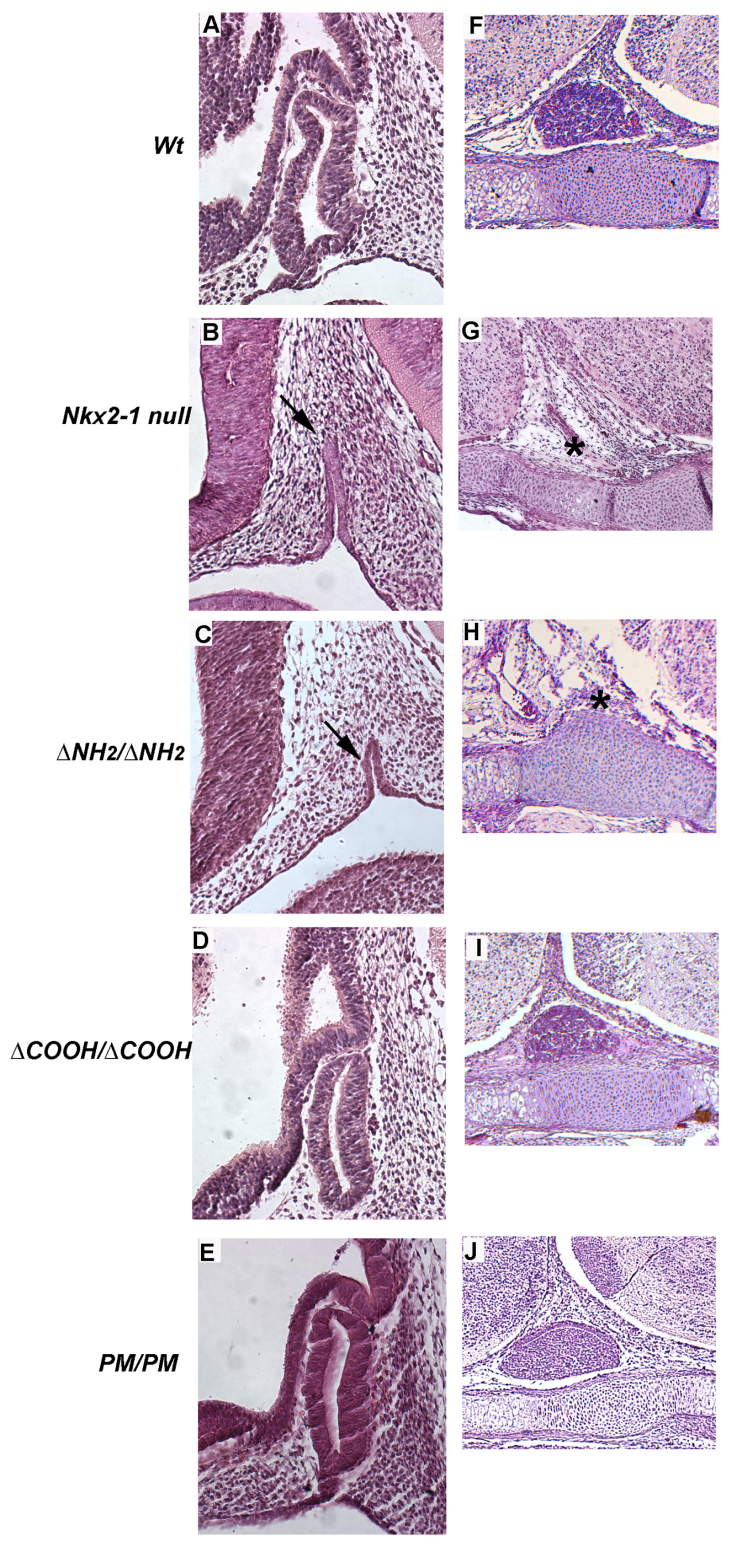
**Pituitary development in Nkx2-1 mutant embryos**. Hematoxylin and eosin staining of sagittal sections from wild type and mutant mouse embryos at E11.5 (panels **A **to **E**) and E16.5 (panels **F **to **J**). ΔCOOH and PM mutants at E11.5 (panels **D **and **E**,) show a Ratke's pouch not different to that of wild type (**A**); on the contrary *Nkx2-1 null* and *ΔNH_2_*embryos show only a rudimentary Ratke's pouch (panels **B **and **C**, *arrows*). At E16,5, the size of the developing pituitary is comparable in wild type(**F**), *ΔCOOH* (**I**) and *PM* mutants (**J**) while *Nkx2-1 null* and *ΔNH_2_*mutant embryos have no pituitary (panels **G **and **H**, *asterisks*). Original magnification ×200 for all panels.

In rodent embryos Nkx2-1 is expressed in the developing neurohypophysis but it is not present in the Ratke's pouch, the precursor of the adenohypophysis[11]. However, in the absence of Nkx2-1 both structures are absent, demonstrating that the presence of this transcription factor is required, either directly or via the generation of inductive signals, in the organogenesis of the entire pituitary[4]. Consistent with this model, at E11.5, in *wt *embryos (Figure 2, panel A), the neuroepithelial cells of the infundibulum are in close association to the anterior wall of the Ratke's pouch. At E16.5 the pituitary appears as a compact structure localized on the cartilaginous primordium of the basisphenoid bone (Figure 2, panel F). In both *ΔCOOH *(Figure 2, panel D and I) and *PM *homozygous (Figure 2, panel E and J) the pituitary is present and appears to be comparable in size with that of the *wt *embryo. On the contrary, in *ΔNH_2 _*homozygous embryos (Figure 2, panel C), pituitary morphogenesis is severely impaired and it is indistinguishable from what observed in *Nkx2-1 null *embryos with only a small rudimentary Rathke's pouch and no evidence of any infundibular recess (Figure 2, panel B). In both mutants, at E16.5, the pituitary is absent (Figure 2, panels G and H).

These data show that for complete pituitary development only activation domain 1 of Nkx2-1 is essential, while both activation domain 2 and phosphorylation are dispensable. No information was collected on the functional differentiation of the pituitary.

### Thyroid development in wild type and mutant embryos

**Figure 3:**
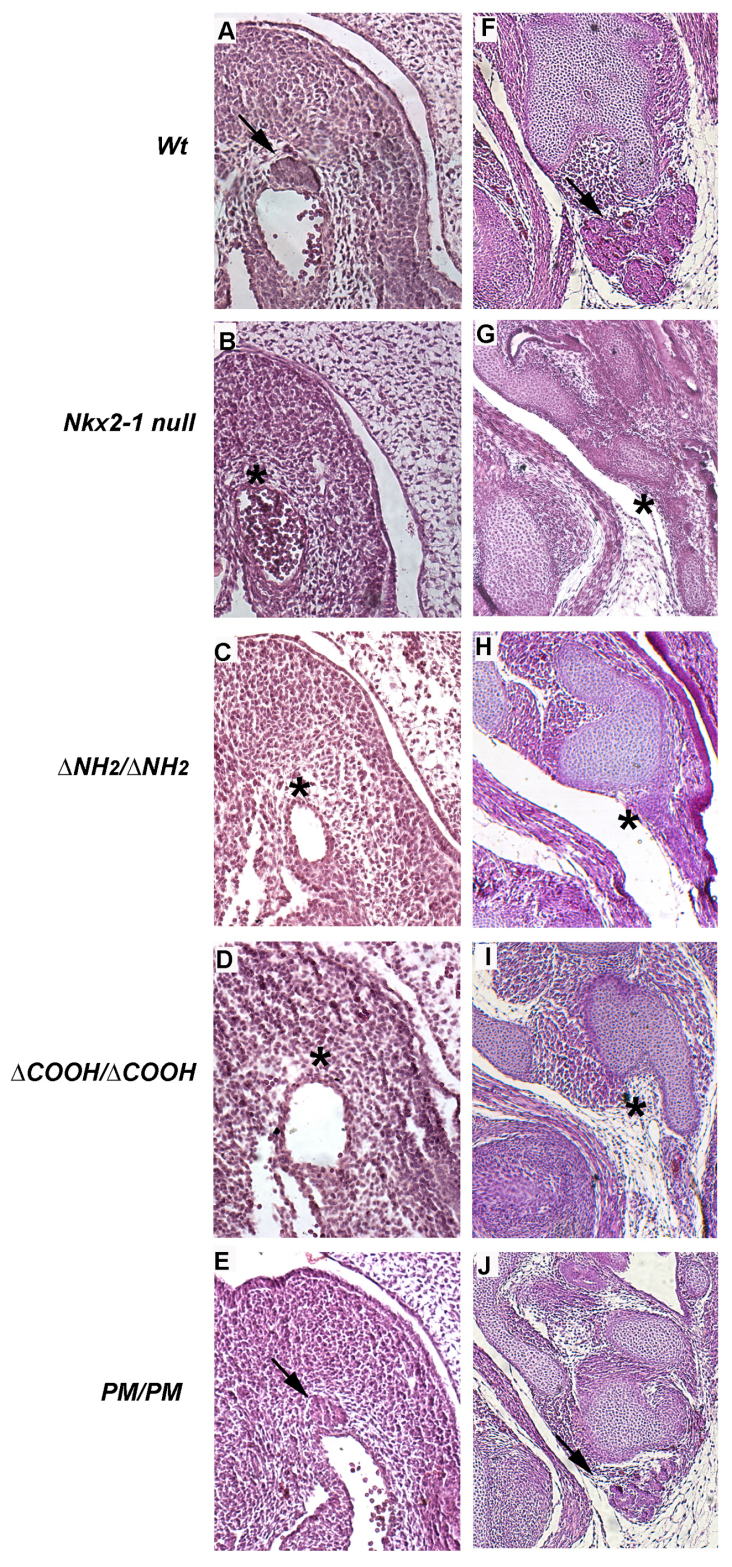
**Thyroid development in Nkx2-1 mutant embryos**. Hematoxylin and eosin staining of sagittal sections from wild type and mutant mouse embryos at E11.5 (panels **A **to **E**) and E16.5 (panels **F **to **J**). At E11.5 *PM/PM *mutant embryos show a thyroid bud (**E**, *arrow*) whose morphology cannot be distinguished from that of the wild type embryos (**A**, *arrow*) while in the other mutant embryos the thyroid bud is not visible (panels **B-D**, *asterisks*). At E16.5, *PM/PM *thyroid is present but appears hypoplastic (compare **J **versus **F**, *arrows*). In *Nkx2-1 null*, *ΔNH*_*2*_and *ΔCOOH* mutants the thyroid is absent(panels **G-I**, *asterisks*). Original magnification ×200 for all panels.

At E11.5 in *wt *embryo the thyroid primordium has lost its connection with the floor of the pharynx, has invaded the underlying mesenchyme and is closely attached to the arcus aortae (Figure 3, panel A)[12]. At this stage, in *Nkx2-1 null *embryos the thyroid bud is undetectable(Figure 3, panel B). The same phenotype is seen in both *ΔNH_2 _*and *ΔCOOH *homozygous embryos (Figure 3, panels C and D respectively). In contrast, *PM/PM *embryos(Figure 3, panel E) show the thyroid bud present, albeit smaller, and correctly located close to the arcus aortae.

At E16.5 thyroid gland has reached its final localization, dorsal to the cricoid cartilage and ventral the trachea (Figure 3, panel F)_. _At this stage, the thyroid is absent in both *null, ΔNH_2 _*and *ΔCOOH *homozygous embryos (Figure 3, panels G, H and I respectively), while in *PM *homozygous embryos the thyroid is correctly localized but it is clearly smaller than the gland of age-matched *wt *embryos(Figure 3, Panels J). Thus, at variance from pituitary, both Nkx2-1 transactivation domains are necessary for thyroid development. The *PM* mutant does not seem to affect early morphogenesis of the gland but it does influence the size of the gland. Thus we decided to investigate in better detail the thyroid differentiation in these mutants.

### Thyroid differentiation in *PM/PM *embryos

**Figure 4:**
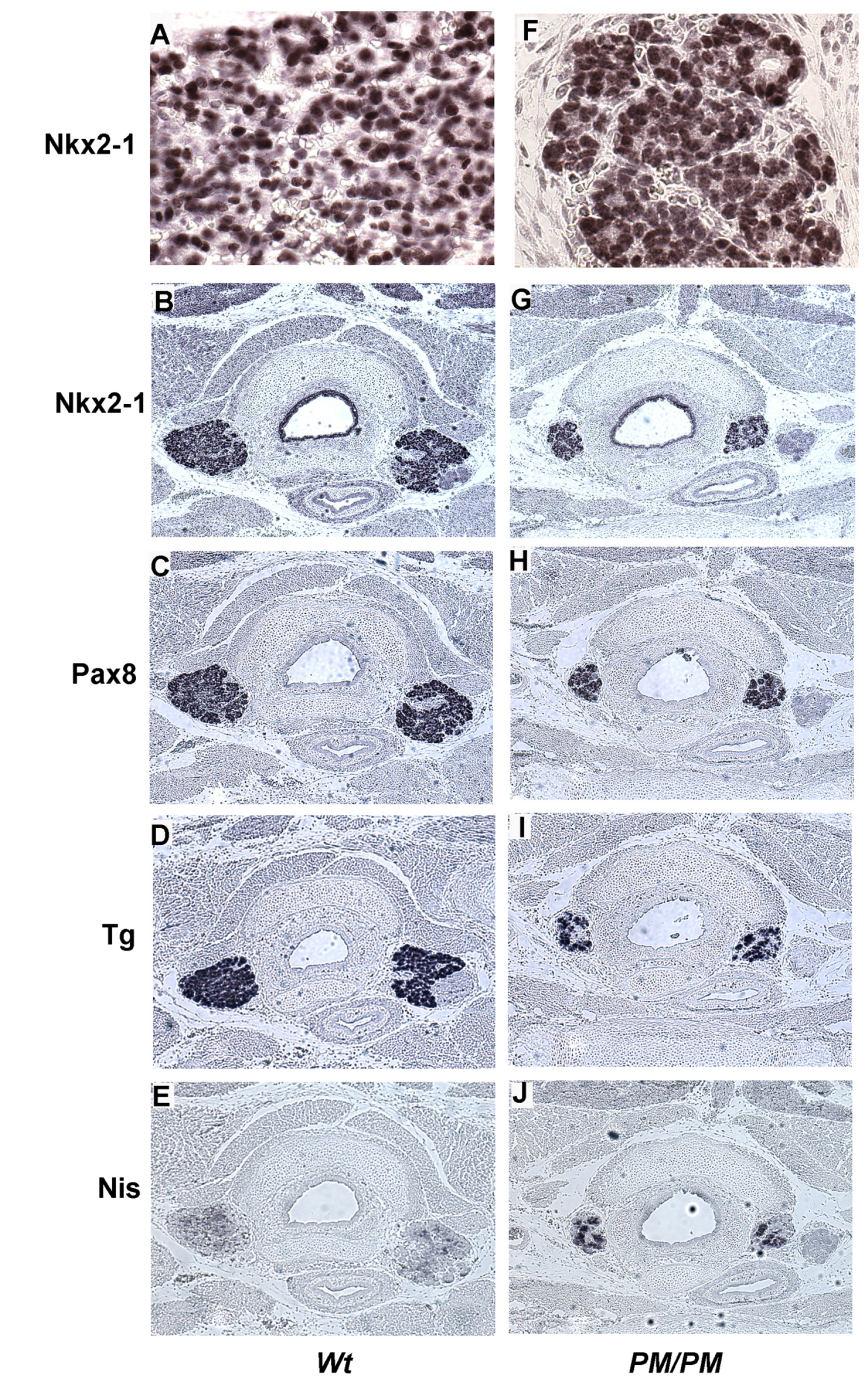
**Thyroid differentiation in E16.5 PM/PM embryos**. Transversal sections of thyroid gland and trachea of wild type (**A-E**) and *PM/PM* (**F-J**) E16.5 embryos were stained with anti-Nkx2-1(**A, B, F **and **G**), anti Pax8 **C **and **H**), anti Tg (**D **and **I**) and anti-NIS (**E **and **J**)antibodies. The size of the *PM/PM* thyroid appears reduced in comparison with the wild type thyroid but the expression of thyroid specific genes is not affected in the mutant gland. Panels **A **and **F **show that the immunostaining for Nkx2-1 is detected in the nuclei of the cells in both wild type and mutant thyroid. Original magnification ×630 for panels A and F; ×100 for the other panels.

At E16.5, the functional differentiation of thyroid has initiated; follicular cells, in addition to Nkx2-1 and Pax8, express Tg and faintly NIS. Immunostaining experiments show that in *PM *embryos Nkx2-1 protein is correctly localized in the nuclei of follicular cells and the intensity of staining is similar between *wt *and *PM/PM *embryos (Figure 4, panels A and B versus F and G). Pax8, Tg and NIS are detected in *PM/PM *thyroid cells, although a decreased number of cells express these proteins (Figure 4, panels C-J). Notably, the expression of NIS appears more robust in *PM *thyroid that in normal gland (Figure 4 panel J *vs*. E).

**Figure 5:**
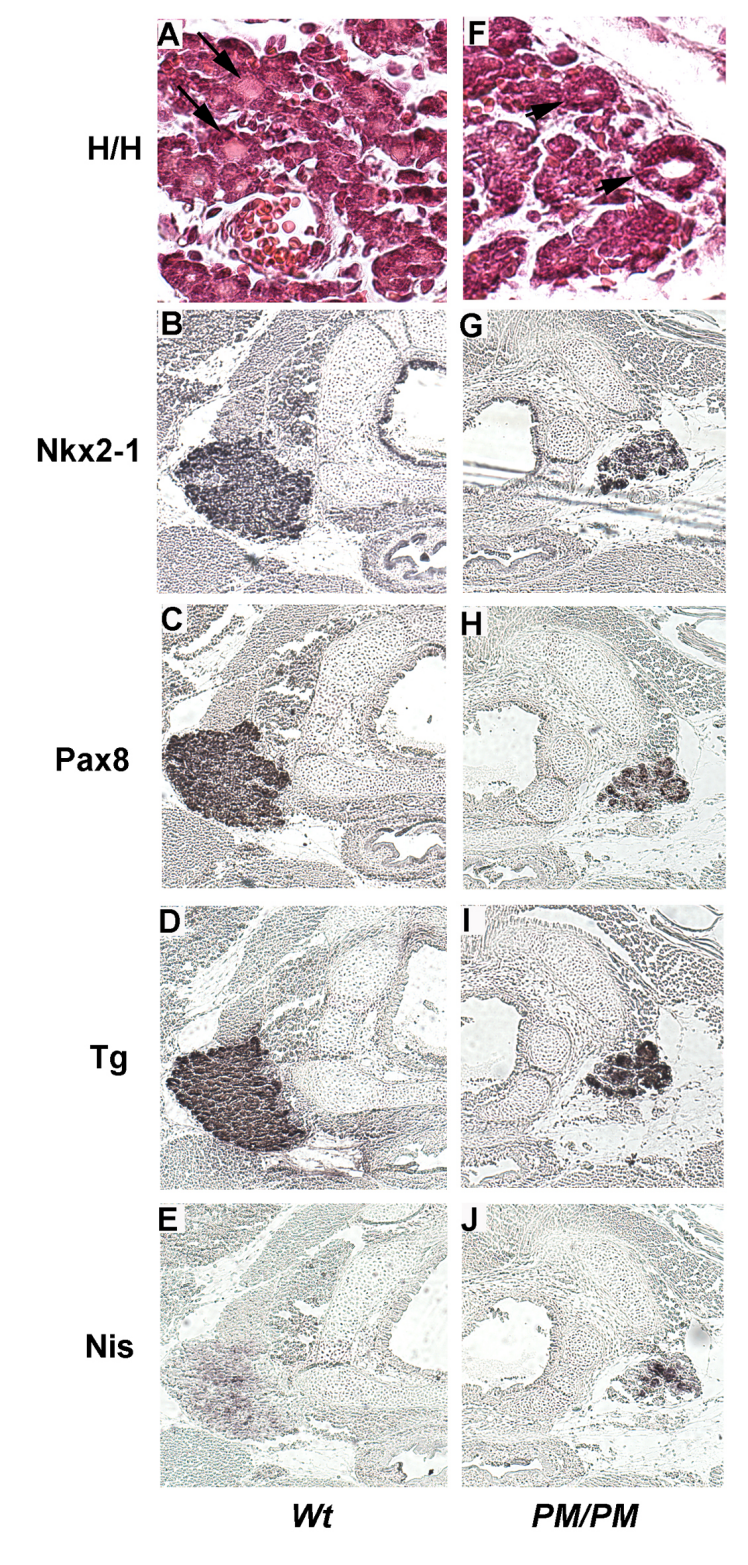
**Thyroid differentiation in E18 PM/PM embryos**. Transversal sections of thyroid gland and trachea of wild type (**A-E**) and *PM/PM* (**F-J**) E18.5 embryos were stained with hematoxylin and eosin (**A **and **F**), anti-Nkx2-1(**B **and **G**), anti Pax8 (**C **and **H**), anti Tg (**D **and **I**) and anti-NIS (**E **and **J **)antibodies. The follicular organization of the thyroid gland (panel **A**, *arrows*) appears impaired and empty follicules are visible (panel **F**, *arrowheads*) in mutant thyroid. In *PM/PM* embryos, follicular cells express Nkx2-1, Pax8 and Tg at levels comparable with those of wild type embryos while the expression of NIS appears increased in the mutant ones(**F **vs **J**). Original magnification ×630 for panels A and F; ×100 for the other panels.

In embryos two days older (E18) thyroid cells of *PM *embryos still express Nkx2-1, Pax8, Tg and NIS (Figure 5, panels B-J) and maintain the ability to synthesize thyroid hormones (data not shown). However, in *PM *embryos the thyroid mass is visibly reduced and folliculogenesis appears to be impaired since only few and empty follicles are present (Figure 5, panel F *vs*. A).

### Nkx2-1 phosphorylation is required for folliculogenesis

**Figure 6:**
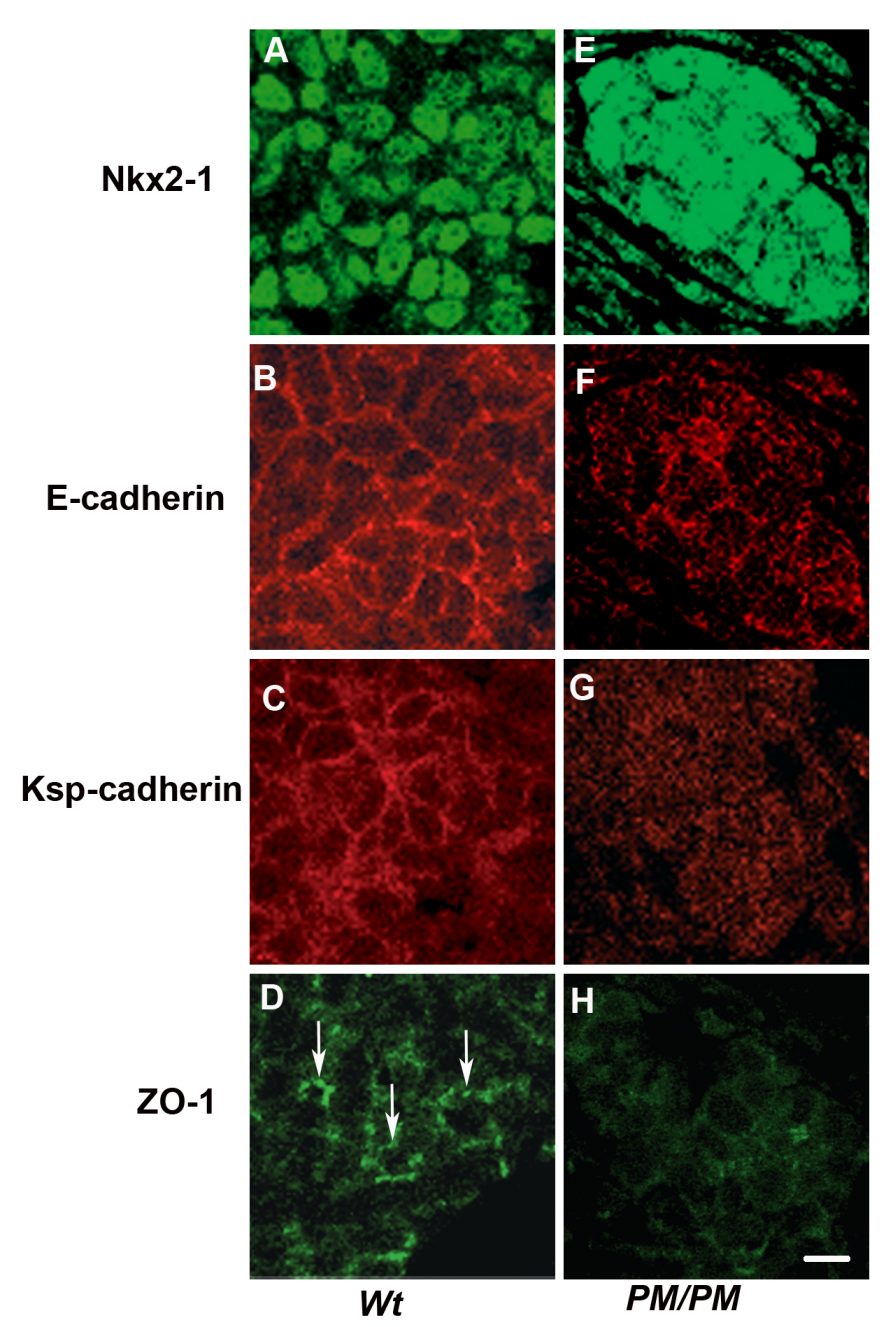
**Expression of E-cadherin, Ksp-cadherin and ZO-1 in developing thyroid**. Transversal sections of thyroid gland of wild type (**A-D**) and *PM/PM* (**E-H**) E14.5 embryos. Double immunofluorescent staining of Nkx2-1 and E-cadherin in wild type (**A **and **B**) and in *PM/PM* (**E **and **F**) mouse thyroids. E-cadherin is present on the plasma membrane of both wild type and *PM/PM* thyrocytes. Thyrocytes are identified by the nuclear staining of Nkx2-1 (**A **and **E**). Adjacent sections were stained by immunofluorescence with anti Ksp-cadherin (**C **and **G**) and anti ZO-1 (**E **and **H**) antibodies. The expression of Ksp-cadherin in PM/PM mouse thyroids(**G**) is significantly reduced with respect to wild type (**C**). ZO-1 is concentrated in spots on the membrane of some cells that possibly surround small lumina (D, *arrows*) while in *PM/PM* thyrocytes there is only a diffuse ZO-1 staining (**H**). Bar 10 μm.

**Figure 7:**
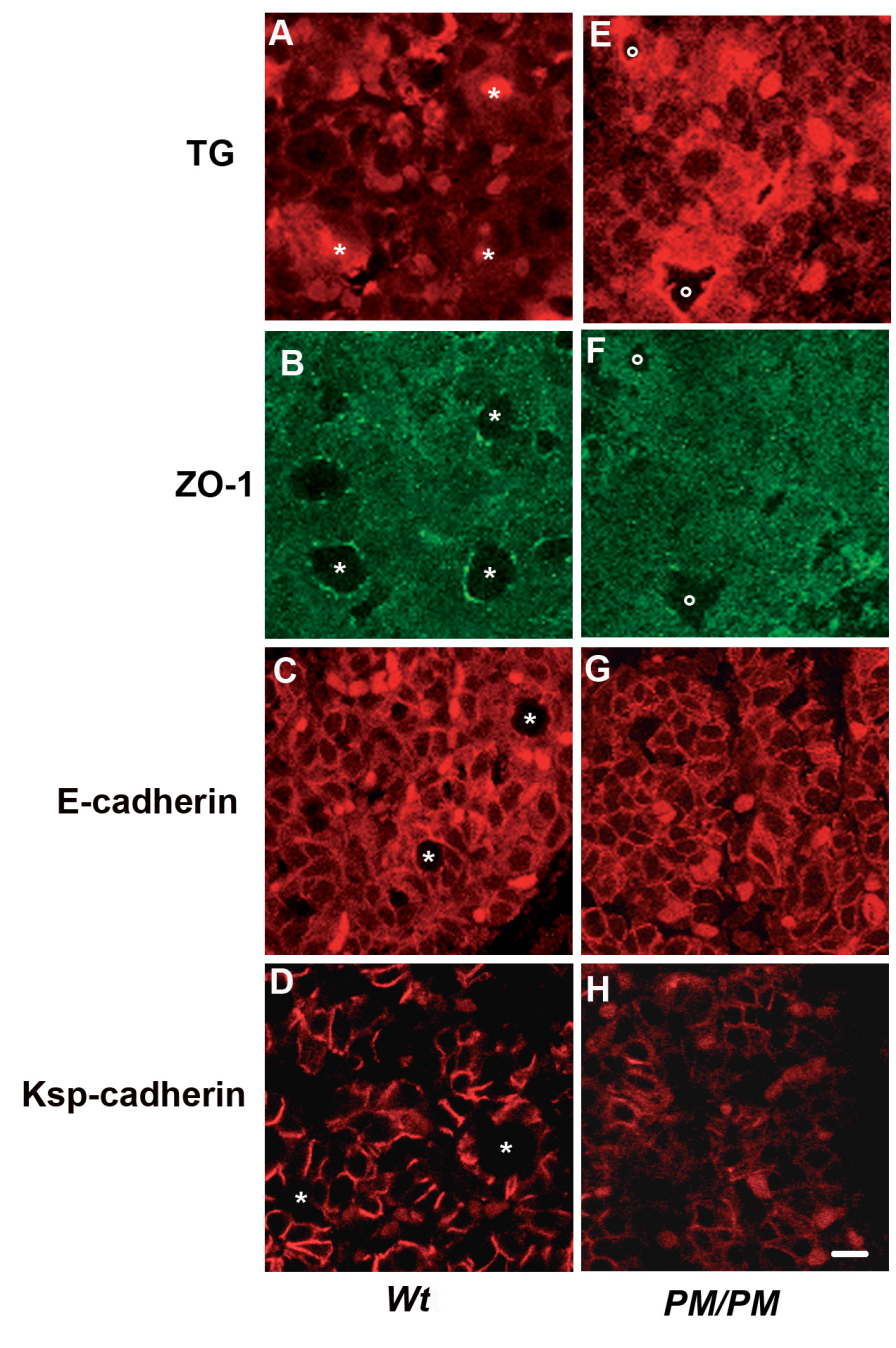
**Expression of thyroglobulin, ZO-1, E-cadherin and Ksp-cadherin in foetal thyroid**. Transversal sections of thyroid gland of wild type (**A-D**) and *PM/PM *(**E-H**) E18.5 embryos. Thyroid from wild type (**A and B**) and *PM/PM *(**E and F**) embryos were stained by double immunofluorescent with anti Tg (**A, E**) and anti ZO-1 (**B, F**) antibodies. In wild type embryos Tg is present in most, but not all, lumina (*asterisk*s) and in the cells. Lumina are defined by the location of ZO-1 at the apical side of the cells. In *PM/PM* thyroids Tg staining is diffused in the gland and is not present in the empty spaces (**E**, *circles*) that do not have characteristics of lumina and have no localized ZO-1 staining (**F**). Wild type (**C **and **D**) and PM/PM (**G **and **H**) thyroids were also stained with anti E-cadherin and anti Ksp-cadherin antibodies. Membrane staining of E-cadherin was observed in both wild type and mutant thyroid (**C **and **G**). The Ksp-cadherin staining was much reduced in PM/PM thyroid (H). *Asterisks *indicate lumina. Bar 10 μm.

To better characterize defects in the follicular organization of the gland, thyroids from *wt *and *PM/PM *embryos, at E14.5 (Figure 6) and E18.5 (Figure 7), were examined by confocal laser microscopy, after immunofluorescence staining, to determine the presence and distribution of the cell-cell adhesion molecules E-cadherin and Ksp-cadherin and of the tight junction component ZO-1. Thyrocytes were identified by the nuclear staining of Nkx2-1 (Figure 6, panels A and E). E-cadherin was present on the thyrocytes plasma membrane of all embryos, at cell-cell contact sites (Figure 6, panels B and F). In the *PM/PM *embryos more intracellular staining was present (Figure 6F) with respect to *wt *embryos (Figure 6B). Ksp-cadherin was expressed in *wt *embryos and was distributed as the E-cadherin (Figure 6C). In mutant mice, a less intense and more diffuse staining was observed and a membrane localization could be barely appreciated (Figure 6G). ZO-1 staining was mostly diffuse over the thyrocytes in all embryos (Figure 6D and 6H). In *wt *embryos there were few areas where ZO-1 staining appeared as intense dots arranged to sorround small roundish areas (Figure 6D) which were suggestive of newly formed lumina.

At E18.5 Tg was well expressed in both *wt *(Figure 7A) and mutant (Figure 7E) thyroids. However, while in *wt *thyroids it was most often concentrated in lumina which were clearly defined by the dotty ZO-1 staining (Figure 7B), in mutant mice no lumina were defined by the presence of ZO-1 (Figure 7F) and Tg, although present in significant amount, was not accumulated in the few irregular empty spaces that were observed (Figure 7E). E-cadherin was similarly located on the plasma membrane of *wt *(Figure 7C) and *PM/PM *(Figure 7G) thyroids, but only in the former lumina were observed and E-cadherin was confined to the basolateral cell domain. Ksp-cadherin was expressed at much higher levels in *wt *thyroids (Figure 7D) where it appeared to be confined to the lateral cell domain of most cells.

Overall these data indicate that the organization of follicular structures with lumina is missing in *PM/PM *mice as indicated by the lack of areas delimited by ZO-1 staining in which thyroglobulin accumulates, like in control thyroids. Lack of follicles correlates with a dramatic reduction in the thyroid/kidney specific Ksp-cadherin. Only in *wt *thyroids, where follicles form, the E- and Ksp- cadherins segregate to the lateral thyroid epithelial cell domain.

### Identification of genes influenced by phosphorylation

The morphology of thyroid gland in *PM/PM mice *indicates that the first steps of organogenesis (i.e. specification of thyroid anlage and migration) as well as the onset of functional differentiation of thyroid follicular cells is unaffected in these mutant mice, whereas events leading to the follicular organization of the gland are impaired. These data suggest a critical role for phosphorylated Nkx2-1 in this latter process. To identify genes whose expression is responsive to phosphorylation of Nkx2-1, RNAs from E18 *PM/PM *thyroids and their wild type littermates were compared using the Affymetrix mouse expression set U74Av2.

**Figure 8:**
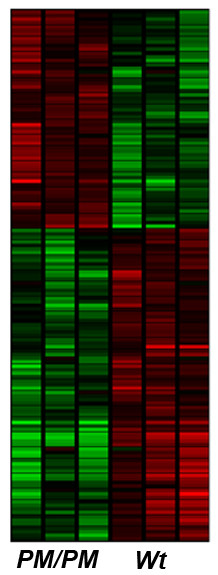
**Two dimensional hierarchical clustering data identifying mRNAs significantly regulated in PM/PM thyroid**. In the two dimensional hierarchical clustering, data from U74 Affymetrix chips identifying mRNAs significantly regulated in E18 PM/PM thyroid in comparison with the wild type are shown. Intensity in the *red *and *green *range indicates the increase and decrease in mRNA abundance, respectively. Each *row *represents a single gene; each *column *represents a particular experimental sample; each *box *represents a normalized gene expression value. Hybridization data from U74 Affymetrix chips were normalized using MAS 5.0 software. For each strain, the data represent the mean of three independent samples each composed of the thyroids dissected from four embryos. In *PM/PM* thyroid, the expression levels of 74 genes were different, with a fold change >2 in comparison with wild type thyroid.

**Figure 9:**
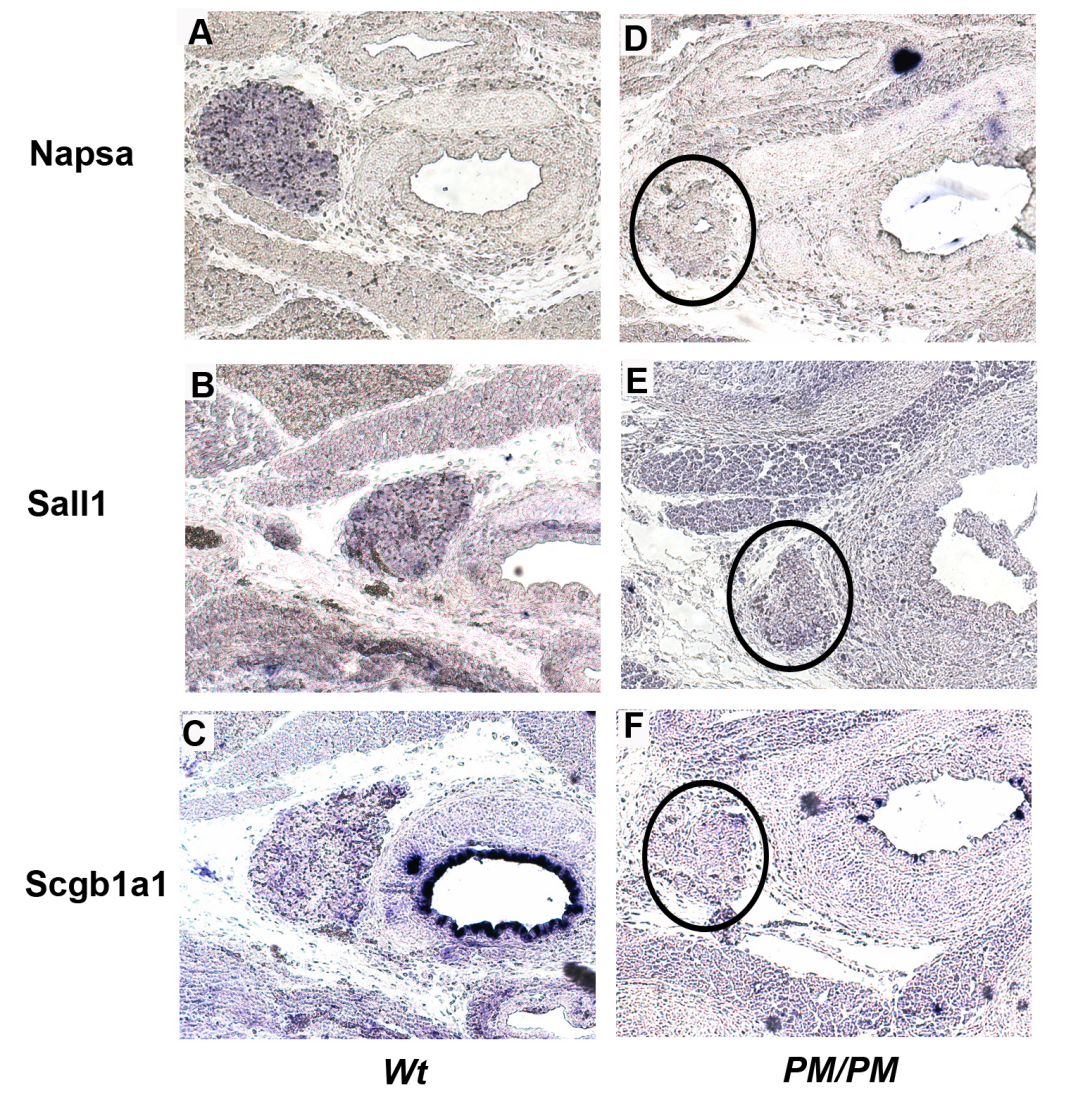
**Expression of some genes in PM/PM thyroid**. Transversal sections of thyroid gland and trachea of wild type (**A-C**) and *PM/PM* (**D-F**) E18.5 embryos were hybridized with the indicated antisense probes, Napsa(**A **and **D**), Sal1 (**B **and **E**) and SCB1a1 (**C **and **F**). In the *PM/PM *thyroid (*encircled*) the levels of expression of these genes appear strongly reduced in comparison with wild type thyroid. These data are in agreement with Affymetrix analysis which indicates that in *PM/PM* embryos Napsa, Sal1 and SCB1a1 are respectively 3.3-, 3.0- and 8.0- fold less abundant than in RNA from PM/PM thyroid. Original magnification ×100 for all panels.

For each genotype, 3 independent RNA samples were used, each representing RNA pooled from 4 embryonic thyroids. Statistically significant differences in the level of gene expression between PM/PM and respective control mice were identified with p-values ≤ 0.1 and fold change ≥ 2.0. Using these parameters, of the 18,000 transcripts with detectable expression in thyroid, the expression levels of 74 genes were different, with a fold change >2. Hierarchical clustering of these differentially regulated genes is shown in Figure 8. 41 mRNAs were decreased (Table 1) and 32 increased (Table 2) in the thyroids from PM/PM fetuses. Three genes, Napsa, Sal1 and Scgb1a1 have been selected for validation by in situ hybridization. Figure 9 shows that in *PM/PM *fetal thyroid the expression of these genes is strongly down-regulated, in agreement with Affymetrix analysis.

**Table 1:** Downregulated genes in *PM/PM *versus *wt *thyroid at E18

Gene Symbol	Gene Title	Fold decrease	Gene onthology category
LOC432613	Trim47	**-11,6**	protein ubiquination
Ager	advanced glycosylation end product-specific receptor	**-11,3**	transport/receptor
Scgb1a1	secretoglobin, family 1A, member 1 (uteroglobin)	**-8,0**	steroid binding phospholipase A2 inhibitor activity
Narg2	NMDA receptor-regulated gene 2	**-7,2**	DNA binding
Calca	calcitonin/calcitonin-related polypeptide, alpha	**-7,2**	hormone
Nkx2-2	Nkx2-2	**-7,1**	regulation of transcription
Iyd	iodotyrosine deiodasi	**-5,8**	
Elovl2	elongation of very long chain fatty acids (FEN1/Elo2, SUR4/Elo3, yeast)-like 2	**-5,5**	fatty acid biosynthesis
Cdh16	cadherin 16	**-4,9**	cell adhesion
S100a5	S100 calcium binding protein A5	**-4,7**	calcium ion binding
Lsm11	U7 snRNP-specific Sm-like protein LSM11	**-4,2**	mRNA processing
3830431G21	RIKEN cDNA	**-4,2**	---
Ero1lb	Ero1-like beta	**-4,2**	electron transport
Klk10	kallikrein 10	**-4,1**	proteolysis and peptidolysis
Acad8	acyl-Coenzyme A dehydrogenase family, member 8	**-4,1**	electron transport
2810055F11	putative proline racemase	**-3,5**	
Napsa	napsin A aspartic peptidase	**-3,3**	proteolysis and peptidolysis
Tgn	thyroglobulin	**-3,3**	thyroid hormone generation
Clic3	chloride intracellular channel 3	**-3,3**	ion transport
Folr1	folate receptor 1 (adult)	**-3,1**	folic acid metabolism
Sall1	---	**-3,0**	regulation of transcription
Tmem213	transmembrane protein 213	**-3,0**	---
Nkd1	naked cuticle 1 homolog (Drosophila)	**-3,0**	calcium ion binding
2410146L05	RIKEN cDNA	**-3,0**	---
6530415H11	RIKEN cDNA	**-3,0**	---
Cldn6	claudin 6	**-2,9**	cell adhesion
Rhof	ras homolog gene family, member f	**-2,7**	GTPase mediated signal transduction
Prdm1	PR domain containing 1, with ZNF domain	**-2,7**	regulation of transcription
Mt1	metallothionein 1	**-2,6**	metal ion homeostasis
Mt2	metallothionein 2	**-2,6**	metal ion homeostasis
Arg2	arginase type II	**-2,5**	arginine metabolism
Nprl2-pending	Tusc4	**-2,5**	cell cycle
Slain1	SLAIN motif family, member 1	**-2,5**	---
Zfp28	zinc finger pt 28	**-2,5**	regulation of transcription
Smarca1	SWI/SNF related, matrix associated, actin dependent regulator of chromatin, subfamily a, member 1	**-2,4**	transcription regulator activity/chromatin modification
Vldlr	very low density lipoprotein receptor	**-2,3**	lipid transport
Rbpms	RNA binding protein gene with multiple splicing	**-2,3**	RNA binding
Transcribed locus	135334_f_at	**-2,1**	---
Atp5j2	ATP synthase, H+ transporting, mitochondrial F0 complex, subunit f, isoform 2	**-2,1**	ion transport
Cited4	Cbp/p300-interacting transactivator, with Glu/Asp-rich carboxy-terminal domain, 4	**-2,0**	transcription coactivator
Zfp26	zinc finger protein 26	**-2,0**	DNA binding

41 genes are downregulated in *PM/PM *thyroid at E18 when compared to wild type. Genes were identified using Affymetrix mouse expression set U74Av2. Statistical significance of p-values ≤ 0.1 and fold change ≥ 2.0 were used as cutoffs for determining expression changes. Genes were categorized based on function determined by gene ontology (GO) term associations.

**Table 2:** Upregulated genes in *PM/PM *versus *wt *thyroid at E18

Gene Symbol	Gene Title	Fold increase	Gene onthology category
Laf4l	lymphoid nuclear protein related to AF4-like	**8,4**	transcriptional activity
Tmc5	transmembrane channel-like gene family 5	**5,0**	ion channel
Adam8	a disintegrin and metalloprotease domain 8	**4,6**	cell adhesion/metallopeptidase
Foxn2	forkhead box N2	**4,0**	regulation of transcription
8430427H17	RIKEN cDNA	**3,5**	
1110003O08	RIKEN cDNA	**3,2**	
Fam183b		**3,1**	
slc5a5	solute carrier family 5 (sodium iodide symporter) member 5	**3,1**	ion transport
Igf2r	Insulin-like growth factor 2 receptor	**2,9**	transport/growth factor binding
4930455F23	RIKEN cDNA	**2,9**	
E230022H04	Ammecr1l	**2,9**	
Rnmt	RNA (guanine-7-) methyltransferase	**2,7**	mRNA capping
Sntb1	Syntrophin, basic 1	**2,7**	actin binding
Krt2-4	keratin complex 2, basic, gene 4	**2,6**	cytoskeleton organization and biogenesis
2410042D21	RIKEN cDNA	**2,5**	
Pvrl3	poliovirus receptor-related 3	**2,5**	cell adhesion
Glt25d1	glycosyltransferase 25 domain containing 1	**2,4**	transferase
1110002H13	Tmem8c	**2,4**	
Zmat5	zinc finger matrin type 5	**2,2**	DNA binding
Dnali1	dynein, axonemal, light intermediate polypeptide 1	**2,2**	cell motility
1110034B05	RIKEN cDNA	**2,1**	
ablim3	actin binding LIM protein family, member 3	**2,1**	regulation of transcription/cytoskeleton organization
Nr1i3	nuclear receptor subfamily 1, group I, member 3	**2,1**	regulation of transcription
UTP6	small subunit (SSU) processome component	**2,1**	RNA processing
Rtn4	reticulon 4	**2,1**	angiogenesis
Ppig	peptidyl-prolyl isomerase G (cyclophilin G)	**2,1**	protein folding
Fam149b	sequence similarity 149, member B	**2,1**	
Wrb	tryptophan rich basic protein	**2,1**	
Gbp7	guanylate binding protein 7	**2,0**	GTP binding
Lmbr1	limb region 1	**2,0**	receptor activity
Rsph9	radial spoke head 9 homolog	**2,0**	cilium axoneme assembly
Akap6	A kinase (PRKA) anchor protein 6	**2,0**	protein kinase A binding

32 genes were dupregulated in *PM/PM *thyroid at E18 when compared to wild type. Genes were identified using Affymetrix mouse expression set U74Av2. Statistical significance of p-values ≤ 0.1 and fold change ≥ 2.0 were used as cutoffs for determining expression changes. Genes were categorized based on function determined by gene ontology (GO) term associations.

Classification of the 73 genes whose expression changes in the *PM/PM *background did not show an enrichment with any specific Gene onthology category. However, the smalll number of genes affected by Nkx2-1 phosphorylation does indicate a specific and restricted pathway controlled by a specific post-translational modification.

## Discussion

We show in this paper that the pleiotropic functions of the transcription factor Nkx2-1 are not a property of the protein as a whole but can be, at least for some of the functions, assigned to specific protein domains or to post-translational modifications. In particular, we focused our attention to the effects of Nkx2-1 in the development of thyroid and pituitary gland using three mutants: a) ΔNH_2_, a mutant deleted of the transcriptional activation domain 1, located at the amino-terminus; b) ΔCOOH, a mutant deleted of the transcriptional activation domain 2, located at the carboxyl-terminus and c) PM, a mutant where serine residues shown to be target for phosphorylation have been mutated in alanines, thus abolishing phosphorylation of the protein. It should be stressed that all these mutants show similar DNA binding and transcriptional activation potential in co-transfection experiments[7,8]. In contrast, we show in this study that each mutant shows a distinct phenotype, revealing a level of complexity that could not be predicted by experiments carried out in cultured cells and stressing the relevance of testing the functions, mostly of proteins with pleiotropic functions, in the context of the whole organism. Knock-out experiments have demonstrated that the absence of Nkx2-1 results in impaired thyroid, lung, brain and pituitary development[4]. In the case of lung, thyroid and pituitary, absence of Nkx2-1 shows absence of the structures where Nkx2-1 is expressed, indicating an essential role of this protein in very early steps of organogenesis. While these experiments showed very clearly the important role of Nkx2-1 in the cell types where it is expressed, the very early disappearance of the cognate structures, in particular of lung, thyroid and pituitary, did not allow any further analysis on the function of the protein later in development. The data provided here demonstrate that one of the functional domain of Nkx2-1, indicated as activation domain 2, is dispensable for pituitary organogenesis. In contrast, both activation domains are required for thyroid development. This observation is relevant in two main aspects. The first is that the search for co-factors interacting with Nkx2-1 for pituitary development could be restricted only to one of the two activation domains. The second is concerned with the debate of whether mutations in either transcription factors or in regulatory region are involved in evolutions of organisms. One argument against a putative role of mutations in TFs in evolution is the pleiotropy shown by most, if not all, this type of regulatory molecules. However, we demonstrate here that the pleiotropic action of Nkx2-1 is due to the combination of diverse functions endowed in different domains of the protein. Hence, mutations in one of these domains would not result in changes in all of its activity, thus making easier to envisage their positive role in organismal evolution [13]

Along these same lines are the data reported here on the role of phosphorylation in NKx2-1 function in thyroid differentiation. We show that mice expressing the PM mutant do develop lungs, pituitary and thyroid gland, at variance from the Nkx2-1 knock-out mouse. The consequences of the PM mutation in lung functions have already been described[10]. We did not address in this study the effect of this mutation in pituitary differentiation, or in specification of diencephalic neurons where Nkx2-1 plays important roles [14]. In the thyroid, PM mutants show a near-normal expression of the genes involved in thyroid hormone biosynthesis. The most relevant phenotype consists in a radically altered follicular architecture, as demonstrated by the deranged localization of E- and Ksp-cadherin[15,16] and of the tight junction marker Z0-1[17], even though Nkx2-1 protein is expressed at normal levels and it is properly located in the nuclei. Interestingly, these results are comparable with those obtained with conditional *Nkx2-1 KO *in thyroid, where also the main defect is the impaired folliculogenesis[18], suggesting that the dephosphorylated Nkx2-1 is a loss of function mutant. Thus, it can also be concluded from these data that the essential functions that Nkx2-1 plays early in development, as shown by the disappearance of thyroid cell precursors in the *Nkx2-1 null *mouse, do not require phosphorylation of the protein. In contrast, at later stages, when follicular cells reorganize themselves into follicles, Nkx2-1 is capable of supporting proper organization of thyroid follicular cells only if phosphorylated. Thus, it appears that Nkx2-1 phosphorylation triggers a switch in the function of this transcription factor at later stages in development. Such a notion is plausible since the the cell-cell and cell-extracellular matrix interactions, and hence the adhesiveness, must be different between the early and migrating thyroid cell precursor and the later, not moving and differentiated, thyroid follicular cells.

## Conclusions

We show in this study that some of the pleiotropic function of the transcription factor Nkx2-1 can be mapped to distinct portions of the protein or to its phosphorylation. This study shows that many functions can be encoded in diverse portions of the same polypeptide chain and provide an example of how to increase the functional potential of a genome without increasing the gene number.

## Methods

### Animals

Animals were kept in an animal house under controlled conditions of temperature, humidity, and lighting and were supplied with standard food and water *ad libitum*. All animal experimentation respected regulations and guidelines of Italy and the European Union. All the experiments with mice described in this paper have been evaluated and approved (internal ID 0907) from the ethic committee "Comitato Etico per la Sperimentazione Animale" (CESA) of IRSG, Biogem. According to Italian law, the project was sent to the National Authorities on the 11th February 2007.

C57BL/6 and CD1 mice were purchased from Charles River Laboratories (Calco, LC, Italy). *Nkx2-1 null *[4] and *PM/PM *[10] mice, have been described.

The colonies of mutant mice were maintained by crossing heterozygous mice with C57BL/6 wild type animals.

### Generation of *ΔNH_2_/ΔNH_2 _*and *ΔCOOH/ΔCOOH *mice

Mouse *Nkx2-1 *gene was isolated from a strain 129/SV mouse genomic library (Stratagene) using a probe corresponding to the 3′-untranslated region of rat *Nkx2-1*. To prepare the targeting vector, a fragment extending from bp 4656 to bp 10443 of the reported mouse genomic sequence (GenBank™ accession no. U19755), containing the entire coding sequence for Nkx2-1, was cloned in pBlueScript.

To prepare ΔNH_2 _targeting vector, a fragment spanning from the translation start site of *Nkx2-1 *(bp 7957) to the end of homeobox (bp 9480) was removed and replaced by the sequence encoding for the amino acid 159 to 372 of the reported rat *Nkx2-1 *sequence[19]. The sequence 5'-CCAC-CAATG-3' was added to provide a ribosome entry site [20]and an ATG codon for translation initiation.

To prepare ΔCOOH targeting vector, the fragment spanning from the translation start site of *Nkx2-1 *(bp 7957) to the end of homeobox (bp 9480) was replaced by the sequence encoding for the amino acid 1 to 221 of the reported rat *Nkx2-1 *predicted protein sequence[19].

In both constructs a stop codon and the simian virus 40 poly(A) sequence were inserted downstream of the coding sequence. The targeting vectors include HSV-tk and PGK-neo cassette for selection of transfected ES cells.

The target constructs were introduced by electroporation in RI ES cells and selected as described[21]. Genomic DNA from neomycin resistance clone was digested with *Bam*HI and analyzed by Southern blotting using as a 500-bp probe from nucleotide 10512 to nucleotide 11042 of the 3′-untranslated region of the mouse *Nkx2-1 *gene (GenBank™ accession no. U19755).

ES cell clones in which the targeting vector had been properly integrated were injected into C57BL/6 blastocysts. Chimeric mice were bred with C57BL/6 mice for germline transmission of the modified allele. The heterozygous *ΔNH_2_/+ *and *ΔCOOH/+ *mice were maintained by crossing heterozygous mice with C57BL/6 1 wild type animals.

### Genotyping

DNA was extracted from yolk sacs or from a piece of tail of the embryos. The tissue was incubated overnight at 60°C with lysis buffer (50 mm Tris-HCl, 100 mm EDTA, 100 mm NaCl, 1% SDS, 0.5 mg/ml proteinase K), and genomic DNA was extracted by adding 0.3 volumes of 6 m NaCl and then precipitated with isopropyl alcohol. To genotype *PM*, *ΔNH_2_*, and *ΔCOOH *mutants the genomic DNA was digested with *Bam*HI and analyzed by Southern blotting using as a 500-bp probe from nucleotide 10512 to nucleotide 11042 of the 3'-untranslated region of the mouse *Nkx2-1 *gene. *Nkx2-1^+/- ^*mutants were genotyped as described[22].

### Histology, immunohistochemistry and *in situ *hybridization

Animals were killed by cervical dislocation. Staged embryos were obtained by dissection of pregnant females. The day at which the vaginal plug was detected was designed as embryonic day (E)0.5. Thyroids and embryos were fixed overnight at 4°C in 4% paraformaldehyde in PBS at pH 7.2, dehydrated through ethanol series, cleared in xylene, and embedded in paraffin, and 7- μm sections were cut. For histological examinations, sections were dewaxed by standard techniques and stained with Harry's hematoxylin/eosin (BDH) according to manufacturer instructions

For immunohistochemistry studies, slides were. were dewaxed by standard techniques and heat treatment to retrieve the antigen sites was performed.

To quench endogenous peroxidases, the sections were treated with 1.5% hydrogen peroxide in methanol at room temperature. The sections were incubated for 1 h at room temperature with blocking solution (3% BSA/5% goat serum/20 mM MgCl2/0.3% Tween 20 in PBS) and then with primary antibodies overnight at 4°C. Staining procedures and chromogenic reactions were carried out according to the protocols of the Vectastain ABC kit protocol (Vector Laboratories). The primary antibodies used were: anti-rat Nkx2-1[11], anti-mouse Pax8[23], anti-human Tg (Dako) and anti-rat NIS[24].

For *in Situ *hHybridization the following clones from Deutsche Ressourcenzentrum für Genomforschung (RZPD) were used to prepare a digoxigenin-labeled probe using DIG-labeling RNA kit (Roche Molecular Biochemicals) from T7 promoter according to manufacturer instructions: IMAGp998N18717 (Scgb1a1), IMAGp998L103622 (Sall1); IMAGp998C05710 (Napsa). Hybridization was performed as described.(Dathan2002)

Histological sections were examined with an AXIOPLAN 2 microscope equipped with Axiocam digital camera (Zeiss). Images were processed using Axion Vision software and edited with adobe Photoshop software.

### Immunofluorescence and confocal microscopy

Tissue sections were deparaffinized and hydrated through xylenes and graded alcohol series followed by antigen retrieval in sodium citrate buffer [0.01 M (pH 6.0)]. Sections were microwaved for 15 min, washed in PBS and PBS containing 0.2% Triton X-100 for 5 min, and incubated for 1 h with blocking buffer. Tissue sections were then incubated overnight at 4 C with primary antibody diluted in blocking buffer, washed in PBS containing 0.2% Triton X-100 for 5 min and PBS, incubated with the secondary antibody for 1 h at room temperature, washed in PBS containing 0.2% Triton X-100 for 5 min and PBS, and finally mounted in PBS/glycerol (1:1).

Immunofluorescence analysis was performed at a confocal laser scanner microscope (LSM 510; Zeiss, Göttingen, Germany). The lambda of the argon ion laser was set at 488 nm, and that of the HeNe laser was set at 543 nm. Fluorescence emission was revealed by BP 505-530 band pass filter for Alexa Fluor 488 and by BP 560-615 band pass filter for Alexa Fluor 546. Double-staining immunofluorescence images were acquired simultaneously in the green and red channels at a resolution of 1024 × 1024 pixels.

The following antibodies were used: mouse monoclonal antibodies anti-E-cadherin (1:100), Rabbit polyclonal antibodies anti-ZO-1 (1:100) and mouse monoclonal antibodies anti-Ksp-cadherin (1:100) were from Zymed Lab. Inc. (San Francisco, CA). Rabbit polyclonal anti-Titf1 antibodies (1:100) were provided by RDL, mouse monoclonal anti-Tg antibodies (1:100) were from NeoMarkers (Fremont, CA-USA). Alexa Fluor 488 or 543 goat anti-rabbit or anti-mouse were from Molecular Probes (Leiden-NL).

### Phosphorylation assay

A pool of at least three thyroids at E18 (or two lungs) of the same genotype were homogenized in 100 μl (500 μl) of Buffer P (50 mM Tris HCl pH 7.5, 400 mM NaCl, 0.1 mM Na_2_EDTA, 5 mM dithiothreitol, 0.01% Brij 35, Sigma protease(P8340) inhibitor cocktail 1 × using a minipotter at 4°C following repeated freeze/unfreeze cycles. After determining proteins (Bio-rad protein assay) 15 μl of homogenate containing 45 μg of total protein were incubated in presence of 200 U of Lambda Protein Phosphatase (New England Biolabs) for 1 hour at 30°C. in presence or absense of phosphatase inhibitors (50 mM sodium fluoride, 10 mM EDTA). 35 μg of total proteins were resolved in a 4 12% Bis-Tris minigel NuPAGE (Invitrogen) at 200 V for 1 hour. Transfer to a PVDF membrane was done with Bio-Rad Mini-TransBlot as described by the manufacturer and western Blot with α-TTF1 (1:10000) were performed as described before[25].

### Bandshift Assay

Cellular extracts were prepared as described before[8]. The binding reaction was carried out in a buffer containing 40 mM Hepes, pH 7.9, 200 mM KCl, 0.5 mM dithiothreitol, and 0.3 mg/ml poly(dI·dC). After 30 min of incubation at room temperature, free DNA and DNA-protein complexes were resolved on a 6% polyacrylamide gel run in 0.5 × TBE (2 mM EDTA, 90 mM boric acid, 90 mM Tris-HCl, pH 8.0) for 2-3 h at 4°C. The gel was dried and then exposed to an x-ray film at -80°C. Oligonucleotide C, used to measure TTF-1 binding activity, has been described [26].

### RNA microarray

The thyroid glands were dissected from E18 embryos, and total RNA was extracted with guanidine isothiocyanate and further purified by the RNeasy minikit (Qiagen). cRNA was generated by using the Affymetrix One-Cycle Target Labeling and Control Reagent kit (Affymetrix Inc., Santa Clara, CA) following the protocol of the manufacturer. The biotinylated cRNA was hybridized to the MG-U74Av2, MG-U74Bv2 and Mg-U74Cv2 Affymetrix DNA chips, containing over 36000 genes and open-reading frames from *Mus musculus *Genome databases GenBank, dbEST, and RefSeq. Chips were washed and scanned on the Affymetrix Complete GeneChip Instrument System, generating digitized image data files. Reactions were carried out in triplicate. Analysis DAT files (images) were analyzed by MAS 5.0 to generate image data files (CEL files). Probe sets summary intensities were generated using the gcrma algorithm of the BioConductor package. The normalization of the data set was performed by quantile-quantile method of the Bioconductor package. We have filtered out all genes having less than 2 present call in all replicate groups in order to obtain a subset of probe sets resulting expressed in at least one condition. Furthermore we have filtered out all genes showing fold change less than 1.5 in whatever comparison. Ones the final data set is generated, robustness of differential expression was evaluated through statistical validation. We conducted ANOVA on the filtered 1406 probe sets using a cut-off p-value of 0.01.

Resulting gene list obtained with the software GeneSpring contain 81 genes showing differential expression in at least one comparison. These were used in clustering algorithms (k-means and hierarchical clustering).

## Authors' contributions

**DS **participated in the design of the study, carried out molecular and in situ hybridization studies. **AR, MS **and **MDF **generated the mice. **PM **and **EA **performed the histological studies. **RS **and **PDL **performed the microarray analysis. **GC **and **LN **carried out confocal studies. **MZ **carried out the EMSA assay. **MZ **and **LN **provided intellectual input to the project. **MDF **participated in the design and in the coordination of the study, analyzed the results and helped draft the manuscript. **RDL **conceived of the study, was responsible for the coordination and supervision of the entire project and prepared the final version of the manuscript.

All authors read and approved the final manuscript.
