# Erratum to: In vivo role of different domains and of phosphorylation in the transcription factor Nkx2-1

**DOI:** 10.1186/s12861-016-0130-0

**Published:** 2016-08-23

**Authors:** Daniel Silberschmidt, Alina Rodriguez-Mallon, Prathiba Mithboakar, Gaetano Calì, Elena Amendola, Remo Sanges, Mariastella Zannini, Marzia Scarfò, Pasquale De Luca, Lucio Nitsch, Roberto Di Lauro, Mario De Felice

**Affiliations:** 1 Stazione Zoologica Anton Dohrn, Villa Comunale, 80121 Napoli, Italy; 2 IRGS, Biogem, Via Camporeale, 83031 Ariano Irpino (AV), Italy; 3 Dipartimento di Biologia e Patologia Cellulare e Molecolare, Università Federico II, Via Pansini 5, 80131 Napoli, Italy; 4 Institute of Experimental Endocrinology and Oncology “G. Salvatore”, National Research Council, Via Pansini 5, 80131 Napoli, Italy

## Erratum

After the publication of this work [1] we became aware that Panel D of Fig. 1 included an incorrect panel. In the original figure for mouse thyroid the +/ΔCOOH lane was duplicated in the +/ΔNH_2_ lane. The correct figure is now included in this document as Fig. 1.

**Fig. 1:**
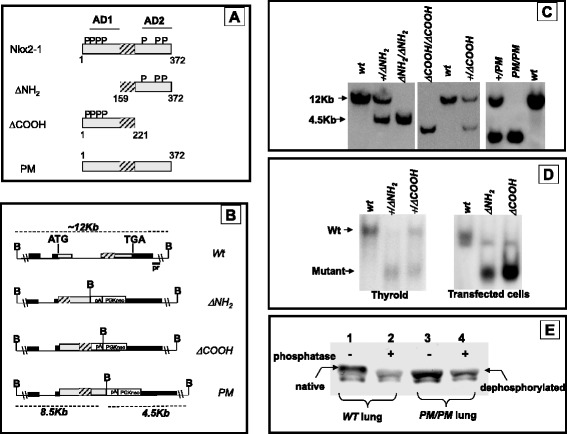
Generation of mice carrying Nkx2-1 mutant alleles. (**a**) The structure of the Nkx2-1 mutants is schematically shown. Numbering of amino acids is shown according to [2]. *P* indicates phosphorylated serine residues according to [3]; AD1 and AD2, activation domains. (**b**) Genomic structure of the *Nkx2*-*1* locus, wild type allele and alleles modified by homologous recombination. *Black boxes* represent exons; *hatched box* the homeobox; ATG and TGA codons are indicated. The probe used for genotyping ES cell clones and mice is indicated by a black bar labeled *pr. PGKneo*, selection marker; *pA*, SV40 poly(A) sequence; *B*, *Bam*HI. (**c**) Southern blot analysis of genomic DNA from mouse tails digested with *Bam*HI and probed probe within indicated in panel **b**. The lower band corresponds to the mutated allele (4.5 kb), the upper band to the wild type allele (12 kb). (**d**) Cellular extract from wild type and mutated mouse thyroids (left) were used in EMSA assays with an oligonucleotide containing a high affinity Nkx2-1 binding site. Extracts from FRTL-5 cells transfected with plasmids encoding mutated forms of Nkx2-1 were used as controls (right). Genotype of the mice and plasmids used in trasfected cells are indicated on each lane. (**e**) Lung homogenates (35 μg of protein) from wild type and PM/PM mice (E18.5) were phosphatase treated (+) or untreated (−), subjected to SDS PAGE, electrophoretically transferred to nitrocellulose and probed with anti Nkx2-1 antibody. The phosphate treatment increases the apparent mobility of wild type Nkx2-1 but does not affect the mobility of PM protein

We regret any inconvenience that this may have caused.
